# Nature relatedness scale: Validity and reliability in the Persian context, factors constructing and influencing it

**DOI:** 10.1371/journal.pone.0274885

**Published:** 2023-04-06

**Authors:** Mohamad Faazeli, Razieh Namdar

**Affiliations:** Department of Agricultural Extension and Education, School of Agriculture, Shiraz University, Shiraz, Fars, Iran; Shenzhen University, CHINA

## Abstract

Psychologically unsustainability stems from the disconnection of humans from the natural environment. Signs of this disconnection have led to the creation of variables generally called “Nature Connectedness” (NC) to measure this relationship. This study is a type of quantitative research, and the method used was a survey. It aimed to investigate the construct validity and reliability of the Nature Relatedness (NR) scale, determine the factors and items constructing, and the variables influencing the NR scale in the Persian context. The NR scale is one of the most used measures in this field and is measured with three factors: Self, Perspective, and Experience. The subjects consisted of 296 students who studied at Shiraz University, School of Agriculture. Based on the construct validity and reliability analysis, the factors and items making up the NR scale were declared valid and reliable (Cronbach’s alpha = 0.86, RMSEA = 0.05). Thus, in this study we deliver one scale for NR which according to validity and reliability indices is appropriate and could be used in future research. SMC values of the observed variables resulting from structural equation modeling showed considerable values. Overall, regression analysis could explain nearly fifty percent in changes of the NR scale through two variables of mindfulness and pro-environmental behaviors. The findings of this research can provide theoretical and practical implications for developing the NR construct. Our findings encourage policies that pay more attention to environmental plans and urban design that promote NC among communities.

## Introduction

Similar to many other countries, the emergence of modernization led to an increase in the extraction of natural resources in Iran through anthropocentric and hegemonic approaches. The urban population in Iran’s first official census in 1956 was about 31.4% of the 19 million population. As a result of the concentration of capital in large cities, lack of job opportunities in rural areas, the growth of services in urban areas, development of industry on city outskirts, and mechanization of agriculture in some rural areas, villagers began migrating to cities and the rural regions were converted into urban ones. Based on the most recent official census conducted in Iran in 2016, approximately 74% of the country’s 80 million people, live in modern cities [1]. Iran currently faces several environmental issues, including unsustainable agriculture, groundwater depletion, drought, soil erosion, land subsidence, and air pollution, all of which harm the country’s natural assets [2–7].

The practice of modernism is linked with the objectification of nature, resulting in the human-nature disconnection [8]. Signs of this disconnection have led to the introduction of measurable indices generally called “nature connectedness” (NC). NC is defined as a sense of appreciation to and affiliation with nature [9] whereby the individual cognitively considers nature to be a part of his self-image [10].

These indices, including the new ecological paradigm [11], connectedness to nature [12], nature relatedness (NR) [9], et cetera., were originally provided for western countries. Since NC has a robust subjective dimension, it varies widely based on individual contexts, especially among different socio-ecological contexts. Insofar as even the term “nature” may be irrelevant for some languages and cultures, it is vital to examine the validity and relevance of NC literature for middle eastern countries, such as Iran [13].

NC can contribute to the health and protection of non-human nature by enhancing pro-environmental behaviors [14, 15]. Pro-environmental behaviors refer to conscious actions to reduce the negative impact of human activities on the natural and built environment [16]. Many researchers and environmentalists have identified it as one of the most critical and influential variables impacting the environment [17, 18].

NC can benefit human well-being in different ways [19, 20], such as improving human cognitive function [21]. A positive relationship with nature is a part of mental well-being even comparable to established factors, including personal income, education, and marital status [22]. NC also is one of the low-cost interventions to enhance people’s well-being [23, 24]. In biology, as well, the biophilia hypothesis explains the inherent biological stimulus that drives humans to connect with nature for survival and fecundity [25].

NC is also associated with mindfulness, one of the essential cognitive capabilities referred to as the third wave approach to cognitive and behavioral therapies in the 21st century [26]. The practice of mindfulness enhances one’s ability to regulate emotions and thoughts, which in turn helps one make better decisions and optimize actions [27, 28]. It is evidenced that the exposure to natural settings can help mindfulness training to be more effective [29]. The correlation between nature and mindfulness consists of a two-way relationship in which both variables reinforce each other [30, 31].

### Persian context

A harmonious relationship with nature has been evident among Iranian indigenous communities. According to Iranian culture, the most lavish celebrations have been based on natural cycles, namely the Nowruz celebration (which represents nature’s freshness and is celebrated on the first day of spring) or the Yalda night (which is the longest night of the year). Iran’s literature has a heritage of praising nature and its elements and using them to express emotions in poetry and prose. Both Islam and Zoroastrianism, the two most influential religions in Iran, respect nature and give advice towards its preservation. Moreover, one of the most accurate solar calendars in the world, the Solar Hijri (Persian) calendar, belongs to the Iranians [32]. The significance of mentioning these cultures and rituals that have already become embedded in people’s psyches is that they can act as forces to encourage individuals to adopt more sustainable lifestyles. However, taking into consideration the other socio-economic factors influencing their behavior is also critical.

Agribusiness is among the most common occupations in Iran. Approximately 4 million people work in the agricultural sector [33]. As agricultural production has increased for greater profits in the past few decades, colossal groundwater depletion has occurred [34], as well as a reduction in soil quality and salinity [35]. Water bankruptcy caused by socio-economic structures is seriously threatening Iran’s natural environment and socio-economic development [36]. Furthermore, agriculture is significantly responsible for global warming and hugely contributes to water pollution globally [37, 38] and locally in Iran [39]. Plowing, spraying, and using pesticides, harvesting and burning agricultural residues, and irrigation with saltwater are among the causes of pollution in agricultural activities.

There is some research that shows the relationship between NC and pro-environmental behaviors of farmers throughout the world [40–42]. In Iran similar studies address the same concerns; for example, two studies demonstrated that farmers’ behavior towards groundwater use is influenced by environmental beliefs [43] and environmental emotions [44]. Along with farmers who play a crucial role in agriculture, the close relationship between agricultural activities and the environment highlights the need for agricultural experts to pay attention to the environment as educators and promoters of sustainable agriculture [45]. However, there is a gap in the literature on carefully studying agricultural experts’ views on nature and sustainability.

### The current research

As current and future experts in sustainable agriculture, students of Agriculture college of Shiraz University were selected to participate in the study. Also, based on a meta-analysis of 77 studies, the prevalence of mental disorders among Iranian university students had an increasing trend from 1991 to 2015, with an overall value of 33.2% [46]. In order to increase their efficiency, universities must address this issue, as the economic losses caused by mental disorders have been estimated to be greater than expected [47]. As mentioned previously, NC improves psychological variables such as well-being and mindfulness. Additionally, Enhancing NC can be expected to provide the basis for a better understanding of oneself, global biological perspective, and experience of nature [9]. Considering the subject’s importance and the weakness of the subject’s background in Iran, we introduced a scale for NC, based on the standard scale of Nisbet et al., and examined its three factors of Self, Perspective, and Experience. Based on a review of previous studies and the selection of key research variables, the following specific objectives are presented:

- Analysis of NR’s three factors (Self, Perspective, and Experience) in the study sample
- Investigating the influential factors (age, psychological well-being, mindfulness, and pro-environmental behaviors) on NR in the study sample.

## Materials and methods

### Participants

The population study included students of Agriculture college of Shiraz University. Students were selected using a random sampling method. According to the number of students of Agriculture college, which was about 1100, and based on Cochran’s formula, the minimum sample size of 285 students was determined for this study with an error level of 0.05. A sample of 296 students finally answered the questionnaire wholly and acceptably. We distributed our online questionnaire to all 11 departments of the college, including Water Engineering, Sustainable Agricultural Extension and Education, Natural Resources, Soil Science, Biosystems Engineering, Plant Production and Genetics, Food Science and Technology, Animal Science, Agricultural Economics, Plant Protection, and Horticulture. Participants were asked about their informed consent in a written form.

### Measures

Table 1 gives an outline of the studies used to design variable indicators. To introduce the NR scale in Iran, the “nature relatedness” scale introduced in 2009, one of the most widely used tools for measuring NC, was used. Like its peer indices, this scale was developed to understand the factors influencing environmentally responsible behaviors because individuals’ identities concerning nature were thought to be closely related to environmental behaviors. Nevertheless, this scale differs from other indices as NR orderly addresses the various factors of the NC concept, namely Self, Perspective, and Experience [9].

**Table 1:** Studies used to design indicators related to research variables.

Variable	Studies used
Nature relatedness	[9, 12, 48, 49]
Pro-environmental behaviors	[50, 51]
Mindfulness	[52, 53]
Psychological Well-being	[54–56]

Self represents an internalized harmony with nature reflected in the thoughts and feelings of an individual’s connection to nature. Perspective indicates a worldview related to nature on a broad scale and considers the behaviors of humans and their impact on all living organisms. The third factor, experience, indicates familiarity and physical affinity with the natural world and the degree of comfort and desire to spend time in nature. The factors “Perspective” and “Experience” each were constructed using five items, and “Self” was constructed using four items (it was initially a five-item factor too, but one item was omitted for reliability issues) with a 5-point Likert scale ranging from strongly agree to strongly disagree. Four out of fourteen scale items were from other NC scales. These will be explained further in the Adaptability to Persian context section. Adaptation to the Persian language and Iranian culture along with face validity was carried out. The items were translated using the back translation method.

There are different well-being measures, but in relation to NC literature, psychological well-being and quality of life are frequently used [8]. In this study, well-being is measured based on the Brief Inventory of Thriving (BIT) scale, which is in the category of psychological well-being [54]. This scale is also confirmed as a valid and reliable measure of psychological well-being in the context of university students in Iran [57]. The Oxford Happiness Questionnaire (OHQ) and Recovering Quality of Life (ReQoL) also contributed to revising the BIT scale according to the current context [55, 56, 58].

For measuring pro-environmental behaviors, Dono et al. study was used as the primary guide [50], which measured pro-environmental behaviors according to Stern et al. and has three subscales: consumer behavior, willingness to pay, and environmental citizenship [59]. For the optimal adaptation in the Persian context, a local scale was used to modify or localize some items [51].

Mindfulness was measured with the Langer Mindfulness Scale (LMS), which has a socio-cognitive perspective on mindfulness and has three subscales: novelty seeking, novelty producing, and engagement [53]. Also, a few indicators of the Brown & Ryan scale, which focus on the role of mindfulness in psychological well-being, were used to provide a more balanced measure of mindfulness in this study [52].

### Procedure

We conducted a cross-sectional survey to collect data. Different departments of the Agriculture college received e-mails and links via social media to complete the questionnaires scored with a 5-point Likert scale from strongly disagree to strongly agree. Before conducting this research, the Higher Education Committee of Agriculture college of Shiraz University and the General Board of Shiraz University Higher Education Committee reviewed and approved the study, and approved the study from an ethical and scientific standpoint. At the beginning of the questionnaires, Participants were asked to consent to the use of their data for scientific purposes. They were assured that their data would be kept confidential.

### Validity and reliability

A panel of researchers and experts in the fields of ecology, psychology, and agriculture examined the face and content validity of the proposed NR scale and other questionnaires. Then, the pilot study was conducted and Cronbach’s Alpha value was calculated to determine the internal reliability of the questionnaires (Table 2).

**Table 2:** Cronbach’s alpha coefficient of questionnaire variables.

variable	Nature Relatedness				Pro-environmental behavior	Mindfulness	Psychological well-being
factor	Self	Perspective	Experience	total	-	-	-
Number of items	4	5	5	14	10	8	10
Cronbach’s alpha coefficient	0.72	0.80	0.78	0.86	0.86	0.69	0.76

### Reliability and validity tests

We used Composite Reliability (CR) and Cronbach’s alpha [60], to assess the internal consistency. CR is an index used to confirm validity [61, 62]; this was calculated for each latent variable by the following formula (Eq 1). Analyzing the CR values showed that all its values were acceptable (CR ≥ 0.7). Furthermore, a panel of experts was assembled to verify the formal validity. Also, to test the construct validity, the average variance extracted (AVE) was utilized (Table 3).


$$P_{c}=\frac{{\left(\sum \lambda \right)}^{2}}{{\left(\sum \lambda \right)}^{2}+\sum \theta }$$ Eq 1

*P_c_*: Composite Reliability

*λ*: Factor Loadings

*θ* = Index error variance

∑ = The sum of each latent variable

Composite Reliability

**Table 3:** CR values and AVE.

Latent Variables	CR	AVE
Self	0.89	0.92
Perspective	0.82	0.93
Experience	0.78	0.80

### Internal consistency of NR factors

The internal consistency of NR factors was analyzed for the validity of this scale. Spearman correlation test was used to calculate the correlation between the factors of NR. As shown in Table 4, all factors had a positive and significant correlation, and the significance level of all correlation coefficients was 0.0001. The factors’ correlations with the total score (ranging from 0.747 to 0.758) are greater than the correlation of every two factors (ranging from 0.346 to 0.445), thus internal consistency was confirmed.

**Table 4:** Correlation matrix of NR factors.

Factor	1. Self	2. Perspective	3. Experience	4. Total
1	1			
2	0.406** 0.0001	1		
3	0.445** 0.0001	0.346** 0.0001	1	
4	0.758** 0.0001	0.747** 0.0001	0.758** 0.0001	1

**Correlation is significant at the 0.0001 level (2-tailed)

### Adaptability to Persian context

In the field of Ecosemiotics, it is explained that language affects our nature experiences [13]. Consequently, if the NC keywords do not match the Persian language, its validity will be seriously questioned. The term “nature” has almost an exact equal in Persian: “طبیعت”, “tabi’at” or “tabi’a.” The common denominator of nature’s definition in the most cited Persian dictionaries of Dehkhoda, Moin, and Amid, as well as in the Oxford and Webster dictionaries in English, is: “What is not human-made”. In both languages, nature is used as the fundamental or inherent features, characters, or qualities of creatures, like “the nature of humans” or “the nature of dogs”, et cetera. Therefore, both humans and non-humans are intertwined in the term in English and Persian. In addition, due to Iranian spiritual traditions, there is familiarity with terms such as connecting with nature and feelings of kinship, appreciation, or unity with nature [32, 63–65].

The current NR scale has ten items from the original NR study [9] and four items from three other significant NC studies [12, 48, 49] that will be mentioned while each item of the three factors is explained. In the factor Self, the items including “connectedness to nature as a part of spirituality”, “considering oneself as a part of nature”, and “a sense of belonging to the natural world as a community” (this item was from Connectedness to Nature Scale, Mayer and Franz [12]) are all systematically familiar in Iran because they are in sync with the compulsory literature and religious courses in high school. About the fourth item, “noticing nature even in urban situations,” although most cities, including Shiraz, should be greener with spatial justice, one can say that the context in which nature can be noticed is partially provided and made possible [66].

In the factor Perspective, the items’ importance has been raised for Iranians more in the new modern era. So the items “Independence of the individual’s well-being from the well-being of the natural world”, (this item was from the Connectedness to Nature Scale [12]) “Humans have the right to use natural resources anyway they want”, and “The need for protection due to nature’s inability to compensate for the human effects” are making sense to Iranians. With increasing awareness of the effects of human activities on animals and birds’ habitats and violations of their rights, the item about “Comparing the rights of animals and plants with humans” is also relevant today. Furthermore, “The impact of individual activities on changing global issues” is a relevant topic for Iranians today with the understanding of concepts like global citizenship.

In the factor Experience, as Badrigargari et al. suggest, “My ideal vacation spot would be a remote, wilderness area” was an item that should be corrected in the Persian version because its factor load was not significant [48]. The item changed to “My ideal vacation spot is in a remote and naturally pristine area.” The result was successful, as stated below, and the item is confirmed. This item, as well as the items of “I enjoy being outdoors, even in unpleasant weather” and “I take notice of wildlife wherever I am,” are among the items that were taken from the original NR scale. “It excites me to watch the changes of plants during different seasons” is a valuable item in any natural setting and added due to the significance of seasons in Iran reflected in the famous season change celebrations like Nowruz, Yalda, and Mehregān, which is also approved in Eslamian et al. study [49]. “I am interested in science documentaries about nature,” which Iranians call the “secret of survival (Raze Bagha)” in informal language, is also decided to be among the items. It is approved as a confirmed item in Eslamian et al. study [49]. According to the latest survey of the Research Center of IRIB Organization, the satisfaction rate of these documentaries is 96% [67]; consequently, they are an integral part of the Iranians’ nature experience. Furthermore, we added this item to the Experience factor because watching these documentaries (audio-visual content in general) is combined with a kind of emotional experience. Although not a completely direct experience with all five senses, today’s brain research–in the NC context–has shown that these audio-visual experiences are equivalent to direct experiences in many respects [68].

### Data analyses

The data was analyzed using SPSS 24 and LISREL 8.8 software. Descriptive analysis of data including average, frequency, mean, standard deviation and ranking were used to describe variables (demographics, mindfulness, NR, pro-environmental behaviors and psychological well-being). Correlation and regression were used in the inferential statistics section. NR had three factors (Self, Perspective, and Experience); the factors’ internal consistency was also examined for the validity of this scale. Correlation test was used to calculate the correlation between the factors of NR. A correlation coefficient test was used to investigate the relationship between variables include NR, pro-environmental behaviors, mindfulness, and psychological well-being.

To analyze the data of NR more thoroughly, factor analysis was carried out using LISREL 8.8 software. Confirmatory Factor Analysis (CFA) verifies and examines the details of the assumed factor structure. We selected the scaled Comparative Fit Index (CFI), scaled root mean squared error of approximation (RMSEA), Standardized Root Mean Residual (SRMR), and scaled chi-square value as measures of fit. The values of the squared multiple correlation (SMC) of NR, also was reported. Finally, a statistical regression test was used to determine the independent variables’ ability to predict NR among Shiraz University students.

## Results

### Demographics

Participants’ average age was approximately 25 years, with a standard deviation of 4.59. The highest frequency was in the age group of 23 to 26 years, and the lowest was among people over 34 years old. The youngest and oldest participants were 19 and 51 years old. The students’ gender distribution showed that of the total sample, 61% (181 students) were female, and 39% (115 students) were male.

The education level distribution showed that the highest frequency was the MSc students (48.3%), and the lowest was the Ph.D. students (19.3%). 264 respondents (89.2%) kept flowers and plants at home or in residence; the rest, 32 students (10.8%), did not keep plants. Approximately 45% of the students’ families owned farmland, garden, or both. More than half of the students’ families did not own any garden or farmland.

### Determining the distribution of students’ perception about NR

The results of Self and Perspective showed that the average was 4.25 out of 5 for Self and 4.41 out of 5 for Perspective, both are very high. For Experience, an average of 3.91 out of 5 is also high (Table 5).

**Table 5:** Descriptive statistics of self, perspective, experience.

Factors	Items		M		SD			Rank
	My connection to nature and the environment is a part of my spirituality		4.22		0.76			3
Self	I am not separate from nature, but a part of nature		4.38		0.70			1
	Even in the middle of the city, I notice nature around me		4.26		0.76			2
	I think of the natural world as a community to which I belong		4.16		0.79			4
	Total mean: 4.25 Range: 1.3–5							
	Humans have the right to use natural resources any way we want		4.38		0.84		4	
Perspective	Conservation is unnecessary because nature is strong enough to recover from human impacts		4.59		0.64		1	
	Animals, birds, and plants have fewer rights than humans		4.28		0.92		5	
	Nothing I do will change problems in other places on the planet		4.40		0.83		2	
	My personal welfare is independent of the welfare of the natural world		4.39		0.80		3	
	Total mean: 4.41 Range: 2–5							
	My ideal vacation spot is in a remote and naturally pristine area	4.03		0.89		3		
Experience	I enjoy being outdoors, even in unpleasant weather	3.11		1.16		5		
	I take notice of wildlife wherever I am	4.12		0.84		2		
	It excites me to watch the changes of plants during different seasons	4.42		0.74		1		
	I’m interested in science documentaries about nature	3.88		0.99		4		
	Total mean: 3.91 Range: 1.4–5							

### Results of confirmatory factor analysis of model

**Fig 1:**
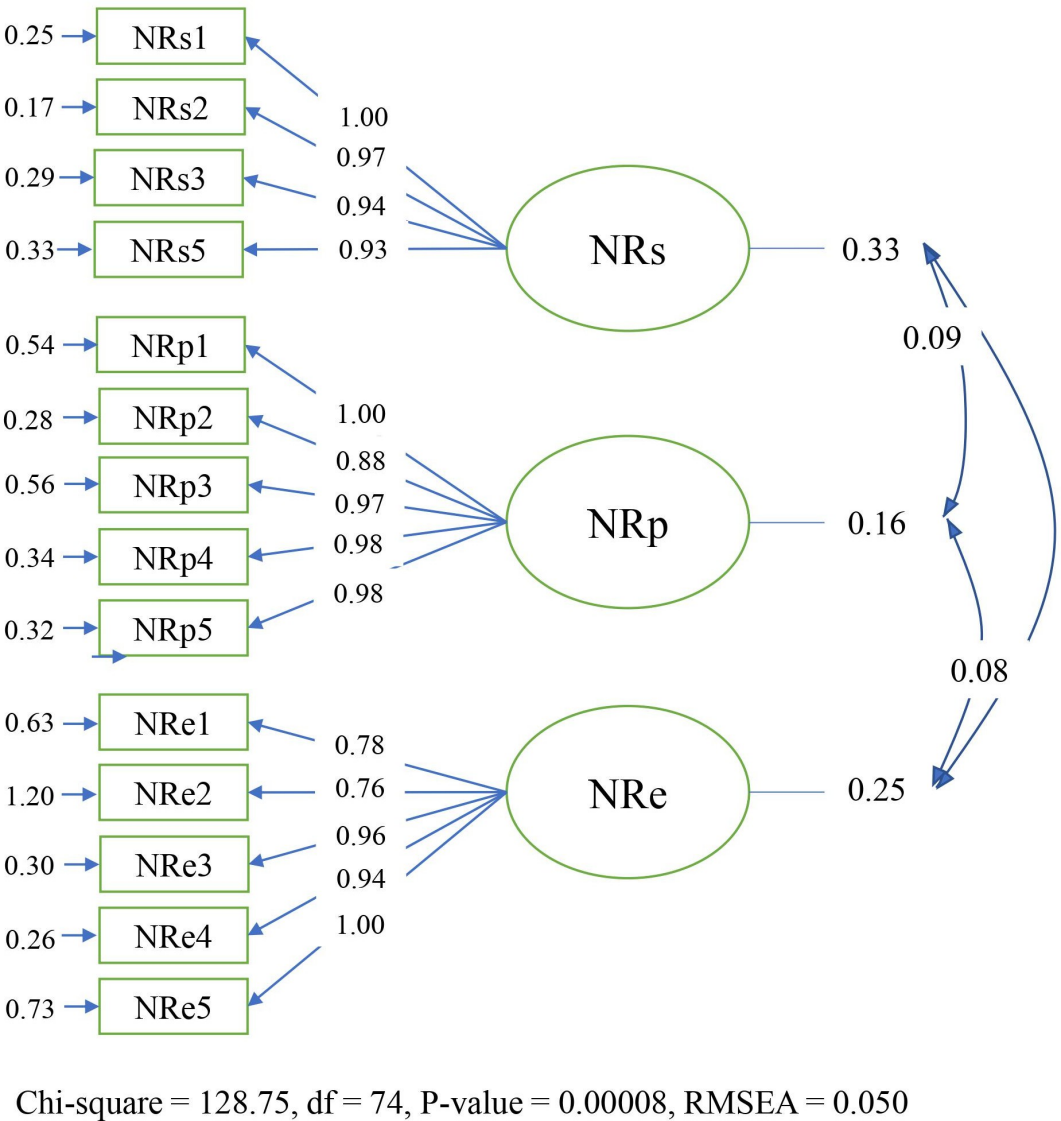
The Path diagram and factor loadings of NR.

As part of the verification and analysis process, CFA examines the details of the assumed factor structure. CFA can test the construct validity and reliability of the indicators (items) that form a latent construct [69, 70]. According to some studies [71, 72], CFA makes it possible for researchers to identify the fitting of the factor structure of a model. Also, to define the factor structure of a scale, CFA gives some indices such as Chi-Square Goodness, Goodness of Fit Index (GFI), Adjusted Goodness of Fit Index (AGFI), Root Mean Square Error of Approximation (RMSEA), Root Mean Square Residuals (RMR), Standardized Root Mean Square Residuals (SRMR), Normed and Non-Normed Fit Index (NFI & NNFI) [73]. The first level of analysis is done from the aspects’ latent construct to the items’ latent construct [69]. Based on the results, all items had a factor load greater than 0.5, and the items of the three factors of NR were approved and remained in the model (Fig 1).

The criterion value (Standard limit) and the reported value of each index named above for the factor analysis model are given in Table 6.

**Table 6:** Standard values and model fit indices.

index	Standard value	Obtained model values
Chi-square / Degree of Freedom (X^2^/df)	3≥	1.85
(NFI) Normed Fit Index	90≤	0.93
Non-Normed Fit Index (NNFI)	90≤	0.96
Comparative fit index (CFI)	90≤	0.97
Goodness of fit Index (GFI)	90≤	0.94
Adjusted Goodness of Fit Index (AGFI)	90≤	0.92
incremental fit index (IFI)	90≤	0.97
Root Mean square Residual (RMR)	0.05≥	0.035
root mean square error of approximation (RMSEA)	0.08≥	0.05

According to Table 6, it can be understood that the goodness of fit statistics for selected constructs is at an acceptable and desirable level. The selected constructs of the research showed acceptable validation in measuring NR significantly.

**Fig 2:**
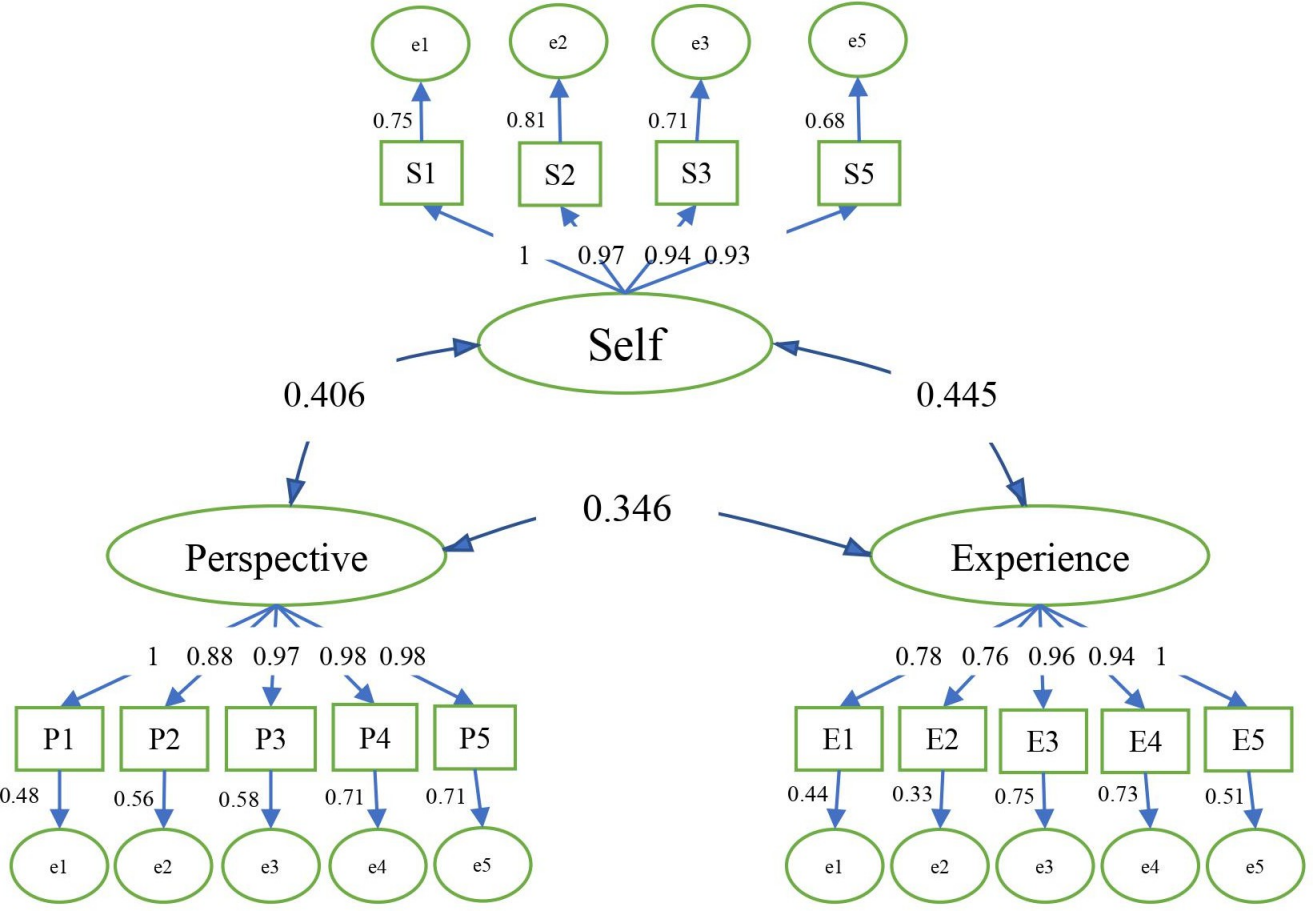
The values of the squared multiple correlation (SMC) of NR.

Table 7 displays an outline of the second-order CFA results. The table illustrates the squared multiple correlation (SMC) values of the observed variables in the output and the optimal reliability of the observed variables. Findings obtained from the factor analysis model showed that among the factors and their items, one item of Self (considering oneself a part of nature), one item of Experience (I take notice of wildlife wherever I am), and another item of Self (considering connectedness with nature as a part of one’s spirituality) had the most effects with path coefficients of 0.65, 0.57, and 0.57, respectively. Two items of Experience (the pleasure of being outdoors even in unpleasant weather and interest in remote and pristine natural areas for an ideal vacation) with path coefficients of 0.11 and 0.19, respectively, had the most negligible impact on NR. In the Fig 2, the numbers on the curve show the correlation between the factors. The numbers on the straight lines show the standard coefficient, and the numbers next to the rectangles indicate the standard error.

**Table 7:** Summary of the results obtained from the second-order CFA.

Variable	Construct	Item	Symbol in the model	Standard coefficient	Standard error	T Value	SMC
Nature Relatedness	Self	Self1	NRs1	1.00	0.75	-	0.57
		Self2	NRs2	0.97	0.81	12.55	0.65
		Self3	NRs3	0.94	0.71	11.24	0.50
		Self4	NRs4	0.93	0.68	10.91	0.47
	Perspective	Pers1	NRp1	1.00	0.48	4.28	0.23
		Pers2	NRp2	0.88	0.56	6.28	0.31
		Pers3	NRp3	0.97	0.58	6.42	0.34
		Pers4	NRp4	0.98	0.71	7.00	0.51
		Pers5	NRp5	0.98	0.71	7.00	0.51
	Experience	Expe1	NRe1	0.78	0.44	5.52	0.19
		Expe2	NRe2	0.76	0.33	4.48	0.11
		Expe3	NRe3	0.96	0.75	7.42	0.57
		Expe4	NRe4	0.94	0.73	7.36	0.53
		Expe5	NRe5	1.00	0.51	-	0.26

### Correlation test results between variables

Correlation coefficient test was conducted to investigate the relationship between NR, pro-environmental behaviors, mindfulness, psychological well-being, and age. As illustrated in Table 8, all variables except age had positive and significant correlations in pairs, and the significance level of all correlation coefficients was 0.0001. The highest correlations were between NR and pro-environmental behaviors (0.423) and mindfulness and psychological well-being (0.404). Age was not significantly correlated with mindfulness; however, it was significantly correlated with NR and Psychological well-being with correlation coefficients of 0.117 and 0.118 at the level of 0.05. Also, at the level of 0.0001, there was a correlation of 0.223 between age and pro-environmental behaviors.

**Table 8:** Correlation matrix between NR and dependent variables.

Variable	1. NR	2. Pro-environmental behaviors	3. Mindfulness	4. Psychological well-being	5. Age
1	1				
2	0.423** 0.0001	1			
3	0.355** 0.0001	0.357** 0.0001	1		
4	0.242** 0.0001	0.322** 0.0001	0.404** 0.0001	1	
5	0.117 0.045	0.223** 0.0001	0.033 0.572	0.118 0.043	1

**Correlation is significant at the 0.0001 level (2-tailed)

### The ability of the independent variables in predicting nature relatedness

Regression test was conducted to determine the independent variables’ ability to predict NR among the students. Age, psychological well-being, mindfulness, and pro-environmental behaviors were considered independent variables and entered the regression model using the enter method. Based on the results, the adjusted value of R^2^ is equal to 0.469. The adjusted correlation coefficient indicates that 46.9% of the changes in the dependent variable are explained by independent variables (Table 9). Analysis of variance with a significance level of 0.0001 also demonstrates the significance of regression and linear relationships between variables (Tables 10 and 11).

**Table 9:** Statistical summary of the fit of the NR model.

Model	multiple correlation coefficient (R)	multiple correlation coefficient (R^2^)	Adjusted coefficient of determination	standard error of estimate
1	0.649	0.526	0.469	0.09744

**Table 10:** Results of one-way ANOVA.

Model		Sum of squares	df	mean squares	F	Sig
1	Regression	0.787	4	0.197	20.722	0.0001
	Residual	2.763	291	0.009		
	Total	3.550	295			

**Table 11:** Coefficients.

Model		Unstandardized Coefficients		Standardized Coefficients	t	Sig
1		B	Std. Error	Beta		
	Constant	0.894	0.068		13.074	0.0001
	X_1_	0.216	0.036	0.340	6.023	0.0001
	X_2_	-0.006	0.037	-0.010	-0.171	0.864
	X_3_	0.204	0.051	0.236	4.030	0.0001
	X_4_	-4.751	0.001	0.000	-0.004	0.997

X_1_ = Pro-environmental behavior

X_2_ = Psychological well-being

X_3_ = Mindfulness

X_4_ = Age

Based on the values in column B, the regression equation can be written as follows:


$$Y=0.894+l0.216(X_{1})+0.204(X_{3})$$ Eq 2

Regression equation of the variables

## Discussion

The predicted NR model had the optimal reliability and SMC of the observed variables. According to the items’ reported value, it was found that the NR model has a suitable and acceptable model fit, and the overall structure of the scale is approved. Further, the evaluation of indices, Average Variance Extracted (AVE), internal consistency, composite reliability, and Cronbach’s alpha demonstrated that the model was reliable and valid. Based on the fitted model of the research, it can be concluded that the factors (Self, Perspective, and Experience) used in this scale, which were collected based on previous studies, can evaluate NR. Overally, the key findings of this study indicated that the structure used to measure NR has acceptable validity and reliability to be used in future research.

The study results reveal that NR-Self is at a high level (with an average of 4.25) among Shiraz University School of Agriculture students. This shows that the subjects feel close to nature, consider themselves part of it, see it with meaning, and find their identity at the cognitive, emotional, and spiritual levels tied to nature. The study results show that NR-Perspective is at a very high level with an average of 4.41, which shows that the subjects pay much attention to nature from a macro perspective and understand human behavior and its effects on other organisms. By examining NR-Experience, it was concluded that the nature experiences of the subjects are at a relatively high level (3.91). The subjects are well acquainted with the natural world and are comfortable and willing to go out in nature [9].

All factors and items can significantly form the NR scale. Based on the indices obtained, the factor Self is the most influential factor among the three factors, and its items have a high share in explaining NR. Findings also indicated that among the items, the second and first items of Self (considering oneself a part of nature and considering connectedness with nature as a part of one’s spiritual identity), and the third item of Experience (I take notice of wildlife wherever I am), respectively, had the most significant impacts on NR.

Based on the results, pro-environmental behaviors and NR were relatively strongly correlated. This correlation is in line with previous research. As some researchers claim, the relationship of NC with pro-environmental behaviors could be due to the relationship of self-image with nature [15]. According to this view, derived from psychology, self-image is a fundamental concept in the human mind that affects habits and attitudes towards nature. Therefore, when nature is not considered apart from oneself, or in other words, when nature is not viewed as outside of one’s identity, concerns and considerations related to this identity are taken into account, and nature is treated responsibly [74]. It should be noted, though, that psychological theories suggest that the reverse of this relationship can also be accurate. Repetition and continuity of behaviors result in a change in self-image and an alteration of identity in line with those behaviors [75]. When a person decides to take care of nature, it may change his personality after some periods of doing it. Thus, as argued, NC does not necessarily take precedence over pro-environmental behaviors, and promoting pro-environmental behaviors, NC can also be increased. Psychologically, both of these variables can affect each other in a cycle and reinforce each other.

Psychological well-being and NR had a moderate positive correlation with each other. This correlation also confirms previous studies on the importance of a positive relationship with nature in psychological well-being. Regarding influence, NR (NC) can be compared with other variables affecting well-being, such as education and income [22]. Also, evidence from some studies suggests that individual differences in NR are associated with differences in well-being [76]. One possible explanation for this could be the biophilia hypothesis in biology, which explains our biological tendency to natural environments and considers it an evolutionary stage in the history of human existence that says humans tend towards environments that further ensure their survival [25, 77, 78].

Strengthening mindfulness could positively affect people’s average NR. This relationship seems to be not just correlational; experimentally, it has been tested in research, and mindful people were more connected to nature [79, 80]. On the other hand, some researchers acknowledge that NC can also lead to mindfulness. The characteristics of natural objects and their specific relationship with sensory receptors and the human brain (such as hearing the sound of nature and smelling flowers) enable sustained attention, which is very useful for improving mindfulness [31, 81, 82].

Regression analysis was used to investigate the causal relationship of variables affecting NR. Findings indicated that two variables of pro-environmental behaviors and mindfulness had a good ability to predict NR. Overall, the independent variables entered in the regression model could explain nearly fifty percent of changes in the dependent variable. Therefore, a significant level of changes in the dependent variable of the research, NR, is predictable.

## Conclusion

Today, the foundations of policy-making in societies have shifted to consider social and environmental sustainability widely. This research was conducted to study and measure NC- which expresses people’s perception of the quality of their relationship with nature- among college students of Shiraz University. In the study sample, nature-relatedness had a desirable level. The supply chain in agribusiness is one of the huge contributors to environmental pollution and consists of many sectors. Agricultural experts influence the way each sector of the supply chain functions. Measuring agricultural experts’ NC can be useful for the design of proper policies and appropriate distribution of human resources.

This NR scale can also be used in the Persian context other than the agricultural sector since the rationale for selecting factors and items is based on public and generalized socio-cultural concerns, as explained. The second-order confirmatory factor analysis of the proposed model for the Persian context indicated that NR could be a function of the three factors of Self, Perspective, and Experience derived from the comprehensive study of Nisbet et al. [9]. All the carefully selected survey items that had been confirmed in terms of reliability and validity (one item was removed from Self due to unreliability) demonstrated their effect on significantly measuring NR. Therefore, the structure of this model is appropriate and shows good compatibility with the research hypothesis.

As two critical reviews suggest recently, NR can be considered a basic psychological need supported by compelling evidence mainly from North America, Europe, Australia, and Asia. However, it is mentioned that there is a gap in evidence from African, South American, and Middle Eastern countries [83, 84]. This study supports the assumption of NR as a basic psychological need and provides evidence for comparing different cultural contexts. The findings of this research can offer theoretical and practical implications for developing the NR construct regarding factors and items.

As the literature in this field acknowledges, it has been found that NC helps to protect the environment by enhancing pro-environmental behaviors and, on the other hand, contributes to the mental well-being of the human mind. Increasing NC is possible by increasing people’s involvement in simple activities such as stopping to see, hear and enjoy the surrounding nature. Therefore, proper design and planning for urban and rural areas and incorporating appropriate programs into the formal education system can positively affect the increase in NC. Given the low cost of these interventions for the well-being of villagers and citizens, policymakers are gradually paying attention to them [85]. In addition, noticing the public media policies and employing environmental experts in this field can significantly be effective in promoting the culture.

Considering the efforts made in Iran for the engagement of ordinary people in pro-environmental behaviors [86], we recommend that the relationship between NR and its factors with pro-environmental behaviors should be explored more to help policymakers and officials make optimal decisions. Instead of direct assertive or aggressive environmental messages, which can cause reverse effects in the environmental behavior of some people [87, 88], enhancing NC can be an appealing alternative [89].

In future research, further clarification can be made by examining the cause-and-effect relationship. For example, according to findings of McEwan et al., interventions and programs that strengthen NR may positively increase well-being [58]. However, coherent studies have not yet been conducted to discover the causal relationship between these variables. Thus, conducting quasi-experimental and educational research in the field of NC, pro-environmental behaviors, psychological well-being, and mindfulness can be very beneficial.

## Supporting information

S1 Data (SAV)
